# Influencing factors and predictive model of postoperative infection in patients with primary hepatic carcinoma

**DOI:** 10.1186/s12876-023-02713-7

**Published:** 2023-04-12

**Authors:** Yanan Ma, Bing Tan, Sumei Wang, Chaoyi Ren, Jiandong Zhang, Yingtang Gao

**Affiliations:** 1 Department of Clinical Laboratory, Nankai University Affiliated Third Center Hospital, Tianjin, 300170 China; 2 Tianjin Key Laboratory of Extracorporeal Life Support for Critical Diseases, Tianjin Institute of Hepatobiliary Disease, Nankai University Affiliated Third Center Hospital, Jintang Road 83, Hedong District, Tianjin, 300170 China; 3 Department of Hepatobiliary Surgery, Nankai University Affiliated Third Center Hospital, Tianjin, 300170 China; 4 Artificial Cell Engineering Technology Research Center, Tianjin, 300170 China

**Keywords:** Primary hepatic carcinoma, Postoperative infection, Risk factors, Predictive model, Nomogram

## Abstract

**Background:**

The purpose of this study was to explore the risk factors for postoperative infection in patients with primary hepatic carcinoma (PHC), build a nomogram prediction model, and verify the model to provide a better reference for disease prevention, diagnosis and treatment.

**Methods:**

This single-center study included 555 patients who underwent hepatobiliary surgery in the Department of Hepatobiliary Surgery of Tianjin Third Central Hospital from January 2014 to December 2021, and 32 clinical indicators were selected for statistical analysis. In this study, Lasso logistic regression was used to determine the risk factors for infection after liver cancer resection, establish a predictive model, and construct a visual nomogram. The consistency index (C-index), calibration curve, and receiver operating characteristic (ROC) curve were used for internal validation, and decision curve analysis (DCA) was used to analyze the clinical applicability of the predictive model. The bootstrap method was used for intramodel validation, and the C-index was calculated to assess the model discrimination.

**Results:**

Among the 555 patients, 279 patients met the inclusion criteria, of whom 48 had a postoperative infection, with an incidence rate of 17.2%. Body mass index (BMI) (P = 0.022), alpha-fetoprotein (P = 0.023), total bilirubin (P = 0.016), intraoperative blood loss (P < 0.001), and bile leakage (P < 0.001) were independent risk factors for infection after liver cancer surgery. The nomogram was constructed and verified to have good discriminative and predictive ability. DCA showed that the model had good clinical applicability. The C-index value verified internally by the bootstrap method results was 0.818.

**Conclusion:**

Postoperative infection in patients undergoing hepatectomy may be related to risk factors such as BMI, preoperative AFP level, TBIL level, intraoperative blood loss and bile leakage. The prediction model of the postoperative infection nomogram established in this study can better predict and estimate the risk of postoperative infection in patients undergoing hepatectomy.

## Background

Primary hepatic carcinoma (PHC) is a malignant tumor with high morbidity and mortality that is prevalent worldwide and seriously endangers human health. Over the past five years, an average of 995,000 new cases of PHC have been diagnosed every year worldwide, of which there were approximately 732,000 cases in Asia, accounting for 73.6% [1]. Hepatocellular carcinoma (HCC) is the most common pathology type, accounting for approximately 75 ~ 85% of cases [2].

Currently, liver resection, liver transplantation, local ablation therapy, and systemic therapy can be used for the treatment of PHC, among which liver resection is the most important method [3]. However, postoperative complications greatly influenced the prognosis of liver resection. Based on multiple clinical analyses, the incidence of postoperative infection is 8.2–20% [4–10]. Prolonged hospital stays, high treatment costs, and even postoperative death can seriously affect the prognosis of patients. Due to impaired liver function, low immune status and other reasons, postoperative infection is prone to occur. According to the site of occurrence, postoperative infection can be divided into abdominal infection, surgical site infection, pulmonary infection, urinary tract infection, etc. The most common type of postoperative complication was abdominal infection, with an incidence of 9.0%, followed by surgical site infection [11]. The causes of postoperative infection after hepatectomy are mainly related to the patient’s primary disease, preoperative systemic state and liver function, surgical scope, and intraoperative blood loss [12]. However, some of these risk factors remain controversial.

Exploring the risk factors for postoperative infection can improve prevention and clinical treatment, reduce or even avoid the occurrence of postoperative infection complications, reduce the time and cost of treatment, and improve the effect and prognosis of patients. Tang et al. found that cirrhosis was a major risk factor for the development of postoperative infection in patients undergoing hepatectomy for HCC [13]. In a case‒control study of 363 patients who underwent HCC resection, Yoshihiro Inoue et al. found no significant differences in infection complications and overall survival between diabetic and nondiabetic patients, which may have been related to their good perioperative glycemic control [14]. Intraoperative blood loss is independently associated with postoperative complications. When intraoperative bleeding exceeds 500 ml, the intestinal mucosa is susceptible to intestinal flora displacement due to ischemia and hypoxia, thus leading to infectious complications [15, 16]. Although some risk factors have been explored in the literature, there are fewer relevant and comprehensive clinical prediction models. In this study, we first performed a multifactorial logistic analysis on the patient’s clinical information and the indications for hepatectomy to identify the risk factors for postoperative infection, and then established a clinical prediction model to analyze the probability of infection more intuitively. The prediction model will assist clinicians in implementing their clinical decisions and allocating medical resources for early clinical intervention and treatment.

## Methods

### Patient selection

We retrospectively analyzed the complete clinical and pathological data of 555 patients who underwent hepatobiliary surgery at our research center from January 2014 to December 2021. According to the inclusion and exclusion criteria, a total of 279 patients who underwent liver resection were selected for the study. They were divided into an infected group and an uninfected group according to whether postoperative infection occurred. Inclusion criteria: (1) Age ≥ 18 years old; (2) Pathological diagnosis corresponding to the “Guidelines for the Diagnosis and Treatment of Hepatocellular Carcinoma (2022 Edition)" [3]; (3) First radical resection of liver cancer; (4) Preoperative liver function classified as Child‒Pugh grade A or B; (5) No preoperative antitumor treatment; and (6) Complete clinical medical records. Exclusion criteria: (1) complicated by other tumors; (2) death within 30 days after surgery; and (3) cases of preoperative infection. Our study was reviewed and approved by the Ethics Committee of the Third Central Hospital of Nankai University, and informed consent was obtained from all the enrolled subjects, who were fully informed of the potential risks and benefits.

### Diagnostic criteria of postoperative infection

In this study, postoperative infections included abdominal infection, surgical site infection, pulmonary infection and urinary tract infection, etc. The diagnostic criteria were determined with reference to the Diagnostic Criteria for Hospital Infections (Trial) [17]. Abdominal infection: purulent discharge from abdominal drainage or puncture, pathogenic bacteria detected in the discharge, or abdominal ultrasound confirmed intra-abdominal abscess. Surgical site infection: purulent discharge from the surgical incision and positive bacterial culture or infection manifestations such as redness, swelling, heat and pain. Pulmonary infection: positive sputum culture or pulmonary auscultation suggesting pulmonary inflammation. Urinary tract infection: symptoms of urinary tract infection and positive urine bacterial culture.

### Perioperative management

Perioperative management is an important part of improving the efficacy and cure rate. Preoperative diagnosis should be improved, and liver function should be correctly assessed. For preoperative coinfection with Hepatitis B virus (HBV), the replication and activation of HBV should be closely monitored. For those with active viral replication, oral antiviral drug therapy is administered throughout the treatment, such as entecavir, tenofovir, or propofol tenofovir. For Hepatitis C virus (HCV)-associated hepatocellular carcinoma, direct-acting antiviral drugs or pegylated interferon alpha combined with ribavirin antiviral therapy should be administered as long as there is HCV-RNA positivity. For combined diabetic disease, blood glucose levels should be strictly controlled and tested [18]. Strengthen intraoperative anesthesia management, correct selection of operation style, reasonable hepatic resection volume, reduce bleeding, and maintain respiratory and circulatory functions. Postoperatively, we should closely monitor the condition, strengthen liver protection and improve supportive treatment to prevent complications. Intravenous drip of second-generation cephalosporins or ceftriaxone 30 min before anesthesia, with additional metronidazole or cephalosporins for possible anaerobic infections. If the duration of surgery exceeds twice the half-life of the antimicrobial drug or if necessary, an additional dose of antibiotics may be administered.

### Surgical treatment

Patients were treated by laparoscopic surgery or open surgery. In the open group, an incision of approximately 15–20 cm in length was made under the right costal margin to block the blood supply around the liver, a precut line was marked approximately 2 cm from the edge of the tumor tissue, the liver parenchyma was gradually cut and separated, the excision line was in the same direction as the scraping steak, and the larger vessels were encountered in the same direction as the vascular line. The wound was treated with electrocoagulation, dressings were used to stop bleeding, a drainage tube was routinely placed, and the abdomen was closed layer by layer. In the abdominal group, a curved incision was made 1 cm below the umbilicus, pneumoperitoneum was established, and then a Trocar (10 mm) and a 30° laparoscope were placed sequentially. The lesion was effectively resected, and the resection procedure was basically the same as that performed for the open group. The liver parenchyma and ducts were separated using an ultrasonic knife. Hemostasis was achieved by electrocoagulation, drainage tubes were routinely placed, and sutures were placed to close each incision.

### Data collection

Thirty-two clinical indicators were selected for this study. The clinical indicators were divided into general indicators, basic diseases, laboratory indicators and surgery-related indicators. General indicators included sex, age and body mass index (BMI). Basic diseases included histories of hepatitis B, hepatitis C, smoking, drinking, hypertension, diabetes, cirrhosis, etc. Liver function was assigned a Child‒Pugh grading score. Laboratory indicators were presurgical indicators, including neutrophil percentage (NEUT%), albumin (ALB), alpha-fetoprotein (AFP), carcinoembryonic antigen (CEA), total bilirubin (TBIL), alanine aminotransferase (ALT), creatinine (Cr), platelet count (PLT), prothrombin time (PT), and hepatitis B virus deoxyribonucleic acid (HBV DNA). Surgery-related indicators included excision method, intraoperative blood loss, perioperative blood transfusion, lymphatic metastasis, tumor size, surgical mode, ascites, bile leakage, pathological type, operation duration, and China liver cancer staging (CNLC). The above data were obtained from the research center’s medical record system, and Excel tables were used to record data and preliminary screening data.

### Statistical analysis

Statistical analyses were performed by R 4.1.3 and SPSS 25.0. Continuous variables that did not conform to a normal distribution were expressed as medians and quartiles and analyzed by nonparametric rank sum test; categorical variables were expressed as number of cases and percentages and analyzed by chi-square test. A least absolute shrinkage and selection operator (LASSO) regression method was used to downscale the clinical information and indicators of patients and screen the variables with nonzero coefficient characteristics. Based on the screened variables, a multivariate logistic regression analysis was used to determine the risk factors, and a predictive model was established. Represented in line graph form. Performance verification was performed on the constructed nomogram, the consistency index (C-index) and area under curve (AUC) were calculated to verify the discrimination of the nomogram model, a calibration curve was drawn to verify the calibration, and decision curve analysis (DCA) was used to evaluate the clinical utility of the prediction model sex. The bootstrap method was used to internally validate the prediction model by repeating the sampling 1000 times, and the C-index was calculated to assess the discrimination. P < 0.05 was considered statistically significant.

## Results

### Clinical features

A total of 279 patients were included in the study. Among them, 48 patients had postoperative infections and were included in the infected group, and 231 patients did not have postoperative infections and were included in the noninfected group. The incidence of postoperative infection was 17.2% (48/279). Among them, the incidence of abdominal infections and infections in other sites was 9.3% (26/279) and 7.9% (22/279), respectively. In this study, cohort was comprised of 210 men and 69 women, and the mean age was 58.86 ± 11.48 years. A total of 178 patients (63.8%) had hepatitis B, 11 patients (3.9%) had hepatitis C, 221 (79.2%) had Child‒Pugh class A liver function, and 240 patients (86.0%) had a pathological diagnosis of HCC. A total of 189 patients (67.74%) received preoperative antiviral drugs, including the anti-HBV drugs entecavir, tenofovir or cifovir or anti-HCV direct-acting antivirals or pegylated interferon alpha in combination with ribavirin. All patients underwent radical hepatectomy, including 35 (12.5%) laparoscopic procedures and 244 (87.5%) open procedures. The study cohort was divided into an infected group (n = 48) and a noninfected group (n = 231) based on the occurrence of postoperative infection.

There were statistically significant differences among 10 indicators between the two groups (P < 0.05), including history of drinking, Child‒Pugh, TBIL, Cr, excision method, intraoperative blood loss, tumor size, surgical mode, bile leakage, and operation duration. The clinical characteristics of the patients in the study cohort are summarized in Table 1.

**Table 1:** Clinical characteristics of the patients included in the study (N = 279)

Variable	Postoperative infection		P value
	Yes(n = 48)	No(n = 231)	
Sex:			0.182
Male	32 (66.67%)	178 (77.06%)	
Female	16 (33.33%)	53 (22.94%)	
Age (year):			0.498
≤ 60	32 (66.67%)	139 (60.17%)	
> 60	16 (33.33%)	92 (39.83%)	
BMI (kg/m^2^):			0.077
≤ 24	12 (25.00%)	92 (39.83%)	
> 24	36 (75.00%)	139 (60.17%)	
History of hepatitis B:			0.483
No	20 (41.67%)	81 (35.06%)	
Yes	28 (58.33%)	150 (64.94%)	
History of hepatitis C:			0.696
No	47 (97.92%)	221 (95.67%)	
Yes	1 (2.08%)	10 (4.33%)	
History of smoking:			0.720
No/abstinence (> 1 year)	30 (62.50%)	135 (58.44%)	
Yes	18 (37.50%)	96 (41.56%)	
History of drinking			**0.020**
No/abstinence (> 1 year)	32 (66.67%)	151 (65.37%)	
Seldom	6 (12.50%)	56 (24.24%)	
Yes	10 (20.83%)	24 (10.39%)	
Hypertension:			0.873
No	34 (70.83%)	158 (68.40%)	
Yes	14 (29.17%)	73 (31.60%)	
Diabetes:			0.945
No	37 (77.08%)	182 (78.79%)	
Yes	11 (22.92%)	49 (21.21%)	
Cirrhosis:			0.216
No	29 (60.42%)	114 (49.35%)	
Yes	19 (39.58%)	117 (50.65%)	
Child‒Pugh:			**0.031**
A	32 (66.67%)	189 (81.82%)	
B	16 (33.33%)	42 (18.18%)	
NEUT%	64.70 [55.10;71.25]	63.90 [56.90;69.75]	0.531
ALB (g/L)	41.45 [37.20;44.70]	43.10 [39.50;45.95]	0.071
AFP (ug/L)			0.103
≤ 100	31 (64.58%)	178 (77.06%)	
> 100	17 (35.42%)	53 (22.94%)	
CEA (ug/L)	2.59 [1.64;3.42]	2.45 [1.67;3.81]	0.888
TBIL (µmol/L)	17.80 [11.38;30.22]	14.30 [11.05;19.10]	**0.036**
ALT (U/L)	39.00 [21.25;85.25]	30.00 [19.50;49.50]	0.064
Cr (µmol/L)	61.00 [54.00;69.50]	70.00 [60.00;81.00]	**<0.001**
PLT (×10^9/L)	187.50 [142.75;239.75]	170.00 [125.50;218.50]	0.136
PT (sec)	12.85 [12.17;13.90]	13.20 [12.60;14.00]	0.071
HBV DNA:			0.568
Negative	33 (68.75%)	171 (74.03%)	
Positive	15 (31.25%)	60 (25.97%)	
Excision method:			**0.014**
Nonanatomical	6 (12.50%)	72 (31.17%)	
Anatomical	42 (87.50%)	159 (68.83%)	
Intraoperative blood loss(ml):			**< 0.001**
≤ 400	16 (33.33%)	177 (76.62%)	
> 400	32 (66.67%)	54 (23.38%)	
Perioperative blood transfusion:			0.585
No	32 (66.67%)	166 (71.86%)	
Yes	16 (33.33%)	65 (28.14%)	
Lymphatic metastasis:			0.289
No	35 (72.92%)	187 (80.95%)	
Yes	13 (27.08%)	44 (19.05%)	
Tumor size(cm):			**0.008**
≤ 4	9 (18.75%)	93 (40.26%)	
> 4	39 (81.25%)	138 (59.74%)	
Surgical mode:			**0.030**
Laparoscopic	1 (2.08%)	34 (14.72%)	
Open	47 (97.92%)	197 (85.28%)	
Ascites:			0.951
No	37 (77.08%)	179 (77.49%)	
Yes	11 (22.92%)	52 (22.51%)	
Bile leakage:			**< 0.001**
No	34 (70.83%)	226 (97.84%)	
Yes	14 (29.17%)	5 (2.16%)	
Pathological type:			0.718
HCC	40 (83.33%)	200 (86.58%)	
Others	8 (16.67%)	31 (13.42%)	
Operation duration (min)	317.50 [240.00;392.50]	265.00 [202.50;327.50]	**0.001**
CNLC:			0.674
I Period	21 (43.75%)	100 (43.29%)	
II Period	14 (29.17%)	80 (34.63%)	
III Period	13 (27.08%)	51 (22.08%)	

Data are shown as number (%) or median (range); BMI: body mass index; NEUT: neutrophil; ALB: albumin; AFP: alpha-fetoprotein; CEA: carcinoembryonic antigen; TBIL: total bilirubin; ALT: aspartate glutamate transaminase; Cr: creatinine; PLT: platelet; PT: prothrombin time; HBV DNA: hepatitis B virus deoxyribonucleic acid; HCC: hepatocellular carcinoma; CNLC: China liver cancer staging. Bold indicates statistically significant values (P < 0.05).

### LASSO regression analysis

**Fig. 1:**
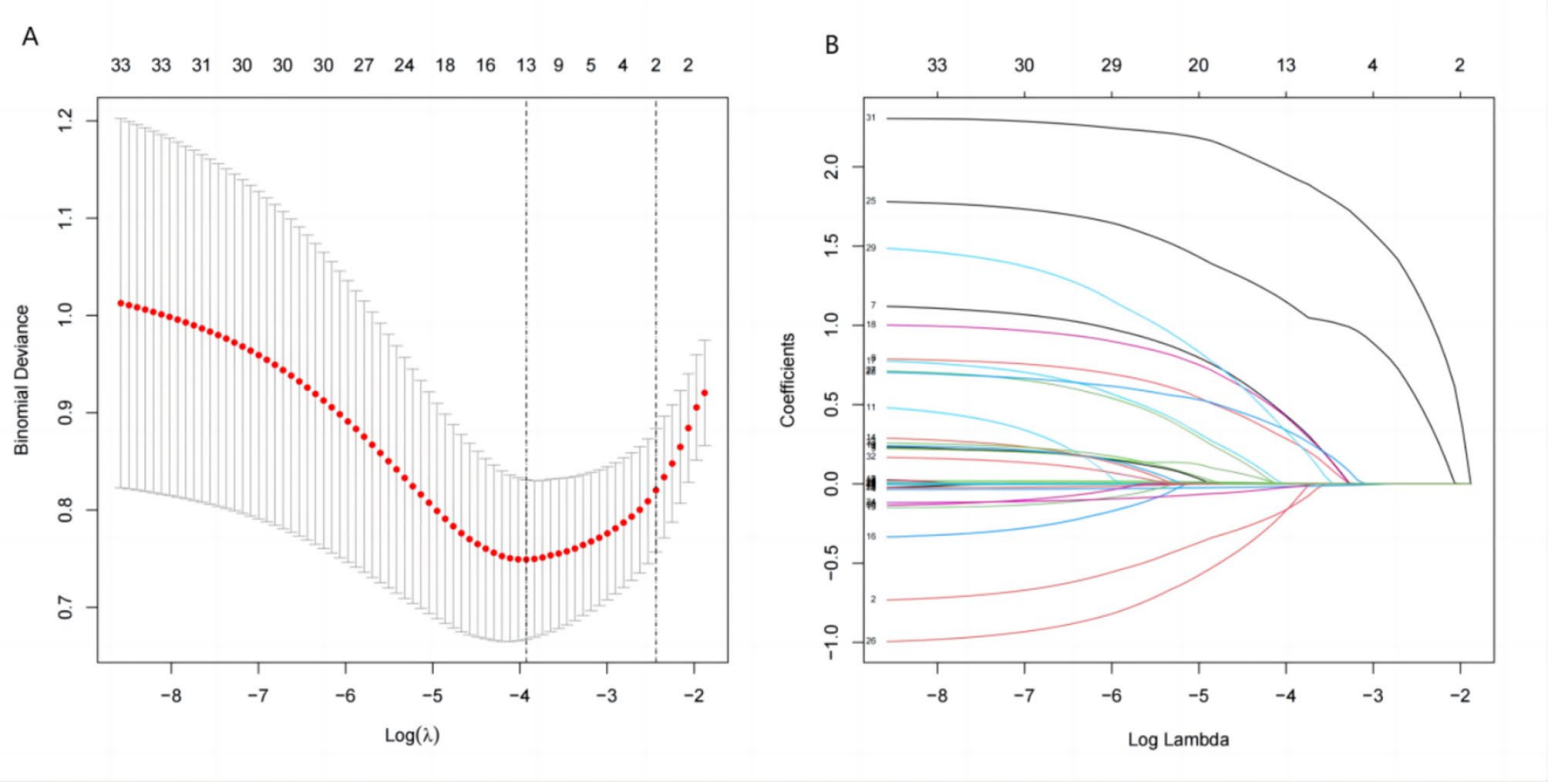
Predictor variable selection based on the LASSO regression method. (a) Optimal parameter (lambda) selection in the LASSO model. (b) LASSO coefficient profiles of the 32 features

Because of the correlation between different independent variables, dimensionality reduction was performed to screen out the most representative predictors of high-risk postoperative infection, and LASSO regression analysis was performed on all independent variables. The analysis results are shown in Fig. 1. Specifically, the parameters are cross-validated, a dotted line is drawn at the optimal parameter (the left dotted line in Fig. 1A), and the corresponding nonzero parameters are 13. At this time, the fitted LASSO regression model is the most suitable. The optimal lambda value screened in Fig. 1A was substituted into the LASSO coefficient curve (Fig. 1B) containing 32 variables in this study, and the intersecting 13 independent variables were the screened nonzero coefficient independent variables. Including age, NEUT%, Cr, BMI, excision method, AFP, TBIL, PT, intraoperative blood loss, perioperative blood transfusion, tumor size, surgical mode, and bile leakage.

### Multifactorial logistic regression analysis

Multifactorial logistic regression analysis was performed on the 13 selected variables, and 5 variables were independent risk factors for infection after liver cancer resection, including BMI (P = 0.022), AFP (P = 0.023), TBIL (P = 0.016), intraoperative blood loss (P < 0.001), and bile leakage (P < 0.001). (Table 2).

**Table 2:** Multivariate logistic regression analysis model for predictors of infection after liver resection

Variable	β	OR (95% Cl)	P value
Age (≤ 60 vs. >60)	-0.548	0.578(0.230–1.368)	0.224
BMI (≤ 24 kg/m^2^ vs. >24 kg/m^2^)	1.056	2.874(1.201–7.525)	**0.022**
NEUT%	0.023	1.024(0.982–1.068)	0.270
AFP (≤ 100 µg/L vs. >100 µg/L)	1.056	2.876(1.154–7.262)	**0.023**
TBIL (µmol/L)	0.007	1.007(1.002–1.014)	**0.016**
Cr (µmol/L)	-0.030	0.970(0.939-1.000)	0.055
PT (sec)	-0.067	0.936(0.768–1.132)	0.483
Excision method (Nonanatomical vs. Anatomical)	0.686	1.986(0.724–6.270)	0.206
Intraoperative blood loss (≤ 400 ml vs. >400 ml)	1.652	5.216(2.032–13.934)	**< 0.001**
Perioperative blood transfusion (Yes vs. NO)	-0.898	0.408(0.140–1.114)	0.089
Tumor size (≤ 4 cm vs. >4 cm)	0.719	2.051(0.789–5.814)	0.154
Surgical mode (Laparoscopic vs. Open)	1.301	3.672(0.601–71.913)	0.241
Bile leakage (Yes vs. NO)	2.475	11.886(3.191–50.597)	**< 0.001**

β: regression coefficient; OR: odds ratio; Cl: confidence interval; BMI: body mass index; NEUT: neutrophil; AFP: alpha-fetoprotein; TBIL: total bilirubin; Cr: creatinine; PT: prothrombin time. Bold indicates statistically significant values (P < 0.05).

### Nomogram prediction model

**Fig. 2:**
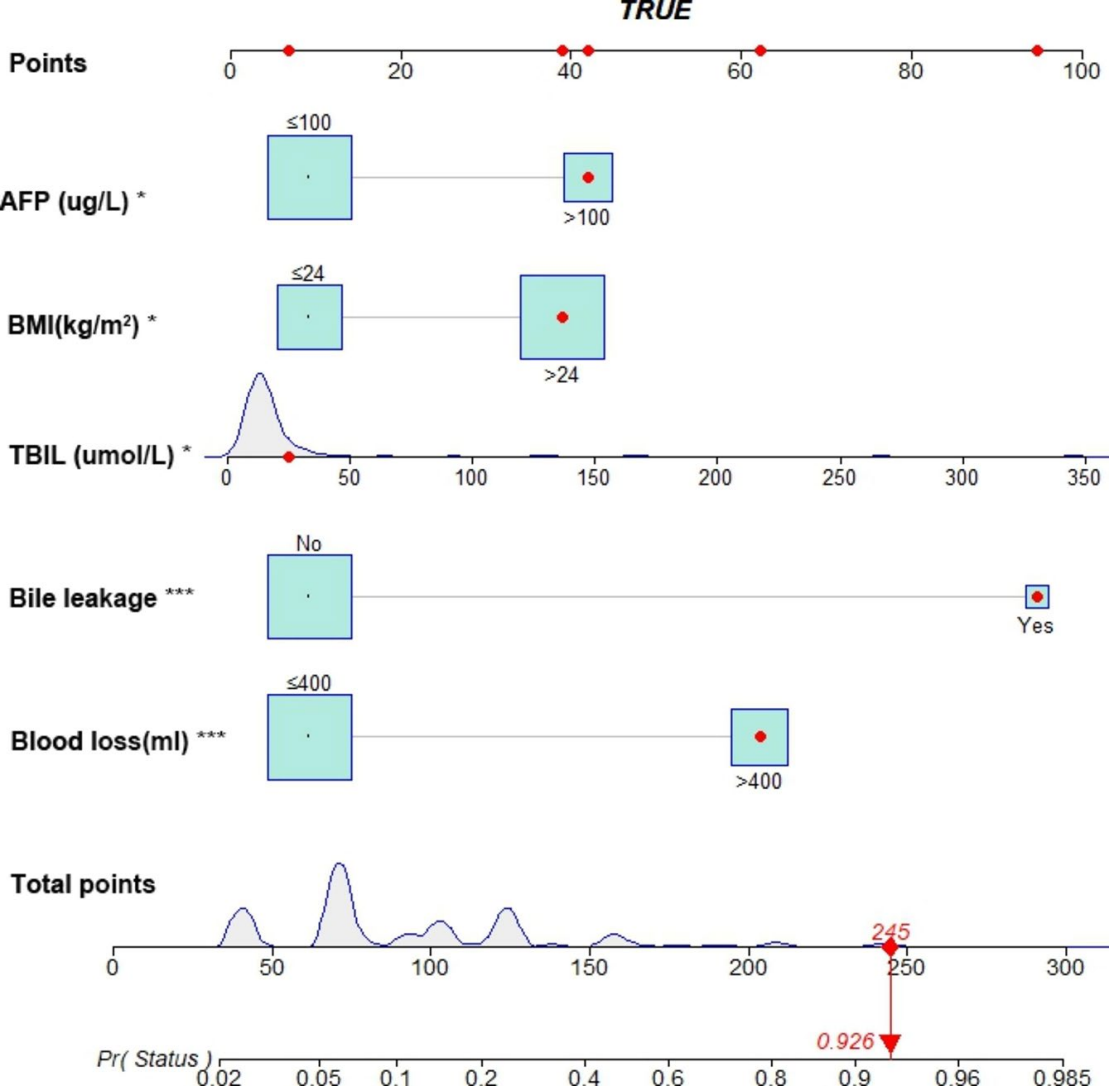
Nomogram prediction model for predicting postoperative infection in patients undergoing liver resection. The red dots are the scores of a true-positive patient according to each of the nomograms, with a final total score of 245 and a predicted probability of infection of 0.926. AFP: alpha-fetoprotein; BMI: body mass index; TBIL: total bilirubin; *: P < 0.05; ***: P < 0.001

Based on the 5 independent predictors, a predictive model for postoperative infection after PHC resection was constructed, and a nomogram was drawn (Fig. 2). The left side of the nomogram is the variable name of the prediction model, the line segment on the right side of each variable represents the range of possible values for the variable, and the “Points” at the top represent the individual scores. The total score of the patient can be obtained by adding the scores of all indicators. A vertical line is drawn downward at the position of the total score, and the intersection point is the probability of postoperative infection of the patient.

### Nomogram Verification

**Fig. 3:**
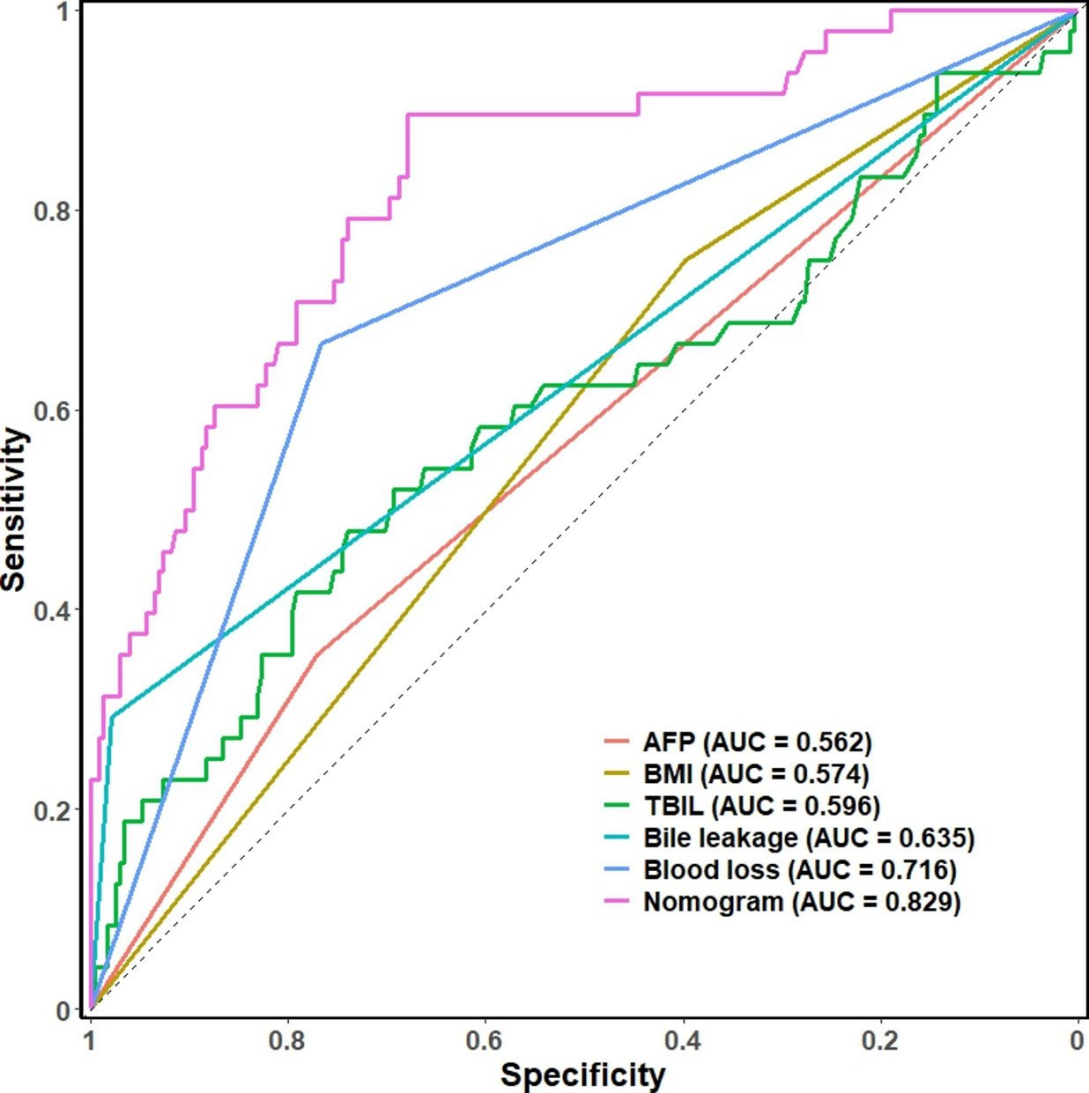
ROC curve of the nomogram for the prediction of infection after liver resection. AFP: alpha-fetoprotein; BMI: body mass index; TBIL: total bilirubin

The C-index of the prediction model was calculated by the R language to evaluate the discrimination of the nomogram. The C-index result was 0.829, which indicated that the constructed nomogram had high predictive ability. The receiver operating characteristic (ROC) curve of each risk factor and the nomogram model was drawn (Fig. 3). The best cutoff value for the nomogram was 0.116, and the AUC was 0.829 (95% confidence interval: 0.680–0.896). It can be seen in the figure that the AUC of the nomogram was higher than that of other single risk factors, indicating that the nomogram prediction model had a high discriminative ability.

**Fig. 4:**
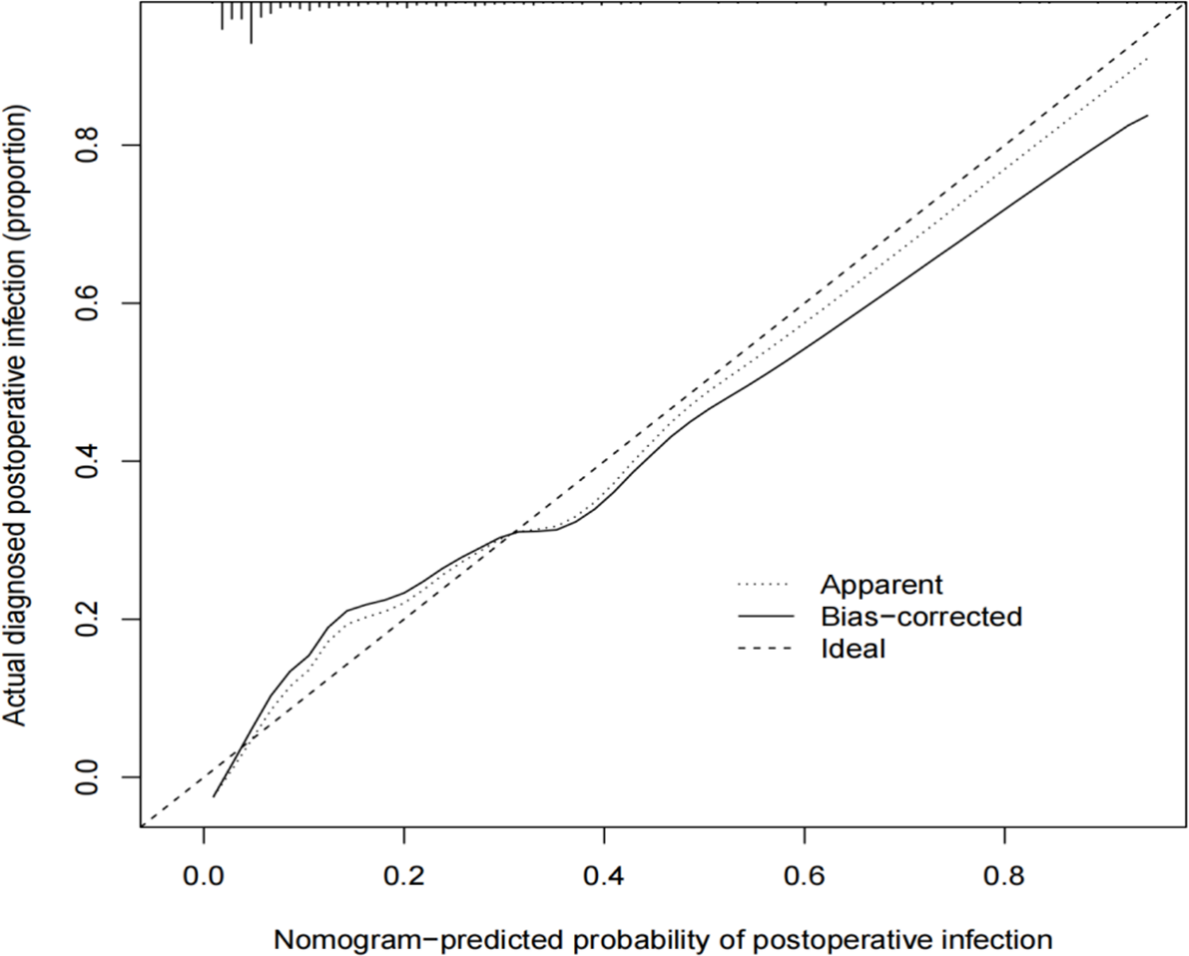
Calibration curve of the infection nomogram after liver resection. The dashed line on the diagonal represents an ideal model, and the solid line represents the performance of the model, where a better fit to the diagonal dashed line indicates a better prediction

A calibration curve (Fig. 4) was drawn to represent the consistent calibration of the nomogram predictions and the actual situation. The abscissa represents the nomogram to predict the probability of infection after liver resection, and the ordinate represents the actual probability. The 45° thick dashed line represents the results of the prediction model under ideal conditions; the thin dashed line represents the consistent calibration results for the entire cohort (n = 279); the solid black line represents the performance of the nomogram. The calculated C-index of the bootstrapping self-sampling method is 0.818, and the predicted probability before and after the correction is consistent with the actual observed probability. The calibration curve of the prediction model fits well with the standard curve, and the degree of calibration is good.

**Fig. 5:**
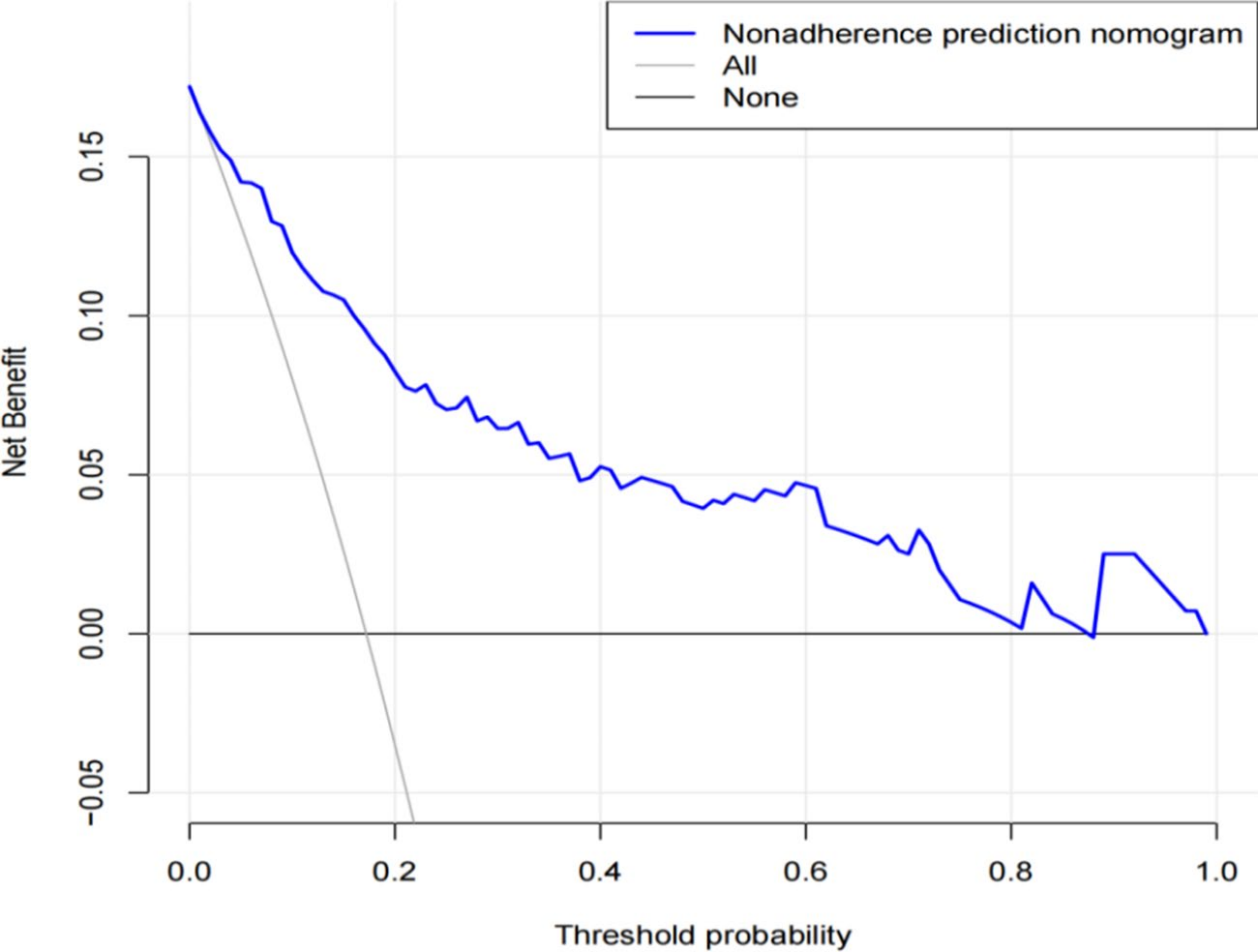
Clinical decision curve analysis of the infection prediction nomogram after liver resection. The blue solid line indicates that using the nomogram to predict the risk of postoperative infection is more beneficial than intervening in an all-patient scenario or a no-intervention scenario

The DCA is shown in Fig. 5. The abscissa is the threshold probability, and the ordinate represents the net benefit. The black horizontal line indicates that all patients had no postoperative infection, and the net benefit was 0; the gray diagonal line indicates that all patients had postoperative infection, and all received treatment. DCA meets the practical need for clinical decision-making by integrating patient or decision-maker preferences into the analysis. The DCA curve shows that the nomogram is more favorable for predicting post hepatectomy infection compared to patients who receive all or no treatment postoperatively.

## Discussion

Hepatectomy is the most effective radical and preferred method of hepatocellular carcinoma treatment. As postoperative care management has improved, the mortality rate of postoperative patients has significantly decreased. However, postoperative infection still seriously threatens the prognosis of patients. Several predictive models have been developed to predict survival and postoperative complications in patients undergoing hepatectomy [19–21]. However, only a few studies have focused on developing models to predict postoperative infections. Multivariate logistic analysis showed that the independent risk factors for infection after hepatectomy were BMI (> 24 kg/m^2^), preoperative AFP (> 100 µg/L), TBIL, intraoperative blood loss (> 400 ml), and bile leakage.

BMI is an indicator of the nutritional status of the body and the degree of obesity, thus directly affecting affect the function of the patient’s immune system and leading to an increased risk of infection [22]. In this study, a BMI > 24 kg/m^2^ was an independent risk factor for postoperative infection in patients with hepatocellular carcinoma, and patients who were overweight or obese had an increased the risk of postoperative infection, similar to the results reported in previous studies [23]. Chang et al. [24] showed that obese patients were more likely to have incisional liquefaction and infection than normal weight patients. Therefore, after hepatectomy in patients with higher BMI, it is important to closely monitor the changes in infection indicators and signs and to whether a postoperative infection has developed.

AFP plays an important role in the diagnosis and treatment of PHC, predicting postoperative recurrence and evaluating high-risk populations. In this study, the multivariate analysis found that AFP was a risk factor for infection after liver resection. Studies have shown that AFP can disable the patient’s anti-infection function by inhibiting immune cells. For example, AFP can inhibit the maturation of DC cells, increase the expression of Caspase-3 and p38-Mark in DCs, induce their apoptosis, reduce the secretion of IL-12, and inhibit the activity of NK cells [25]. Studies have found that AFP inhibits the immune function of NKT cells by downregulating the expression of CD1d [26]. Another study showed that AFP can act on macrophages and reduce their phagocytic activity and the expression of Ia antigen [27].

Hepatic bile duct obstruction, bilirubin metabolism and excretion are blocked, and intrahepatic cholestasis causes hyperbilirubinemia. Ozgen found that hyperbilirubinemia (> 15 mg/dL) was an independent risk factor for postoperative infection [28]. Bile excretion is blocked, microbial colonization occurs, and infection occurs when bile leakage occurs postoperatively [29]. In patients with obstructive jaundice, intestinal bacteria overgrow and are translocated into the systemic circulation. Excessive accumulation of bile acids can lead to impaired liver immune function, thus causing postoperative infection [30].

Tousif et al. found in a retrospective study that intraoperative blood loss is an independent risk factor for postoperative infection [15]. Yanjie et al. noted that intraoperative blood loss affects the incidence of postoperative infection. The risk of infection in patients with blood loss greater than 500 ml is 3.32 times higher than in patients with blood loss less than 500 ml [31]. Intraoperative blood loss leads to liver ischemia, which further leads to structural and functional damage to hepatocytes and affects their metabolic and immune functions.

Postoperative bile leakage has an incidence between 3.6% and 10.0% and is one of the most common complications after hepatectomy [32]. Surgical indications, preoperative treatment and surgical operations have an impact on the occurrence of bile leakage. In a retrospective analysis of 879 patients who underwent hepatectomy, Chikara et al. found that bile leakage was the strongest risk factor for a postoperative organ (or lacunar) infection [33], so, bile leakage leads to poor drainage of infectious bile, which leads to cholestasis and secondary infection [34]. Therefore, the operator should strictly follow the operating procedures and handle the operation with care, and in case of accidental injury, it must be treated immediately. Before closing the abdomen, repeatedly confirm whether there is active bile leak. Active drainage of bile leak can effectively reduce the occurrence of postoperative infection.

Because of the lack of specific clinical manifestations in the early stages of postoperative infection, it is clinically important to evaluate postoperative patients as soon as possible and implement effective interventions in a timely manner. A nomogram is a statistical model for an individualized risk prediction analysis of clinical events based on a logistic regression model, which quantifies the risk of occurrence of clinical events by various risk factors and presents the relevant risk factors graphically, allowing easy visualization of the risk probability values of clinical events [35]. In this study, logistic regression models were constructed based on the results of lasso variable screening. A line graph was constructed to predict the occurrence of postoperative infections by using the five risk predictors of BMI, preoperative AFP, TBIL, intraoperative blood loss, and bile leakage. The prediction model can identify patients at high risk of postoperative infection and help clinicians provide timely and effective interventions for postoperative infection, especially for patients with poor physical tolerance and antimicrobial susceptibility. The ROC curve and C-index were used to verify the discriminatory degree of the model, the calibration curve was used to verify its calibration degree, and DCA was used to demonstrate its clinical effectiveness, which fully validated the good predictive performance of the prediction model. The internal validation results using the bootstrap method showed that the model had a high degree of discrimination. It is presumed that the column line graph model developed in this study has high potential for clinical application in predicting postoperative infection in hepatocellular carcinoma.

Patients with risk factors that have been identified in the model should be given adequate reminders by clinicians. The patients’ systemic nutritional statuses and vital organ functions should be comprehensively assessed preoperatively, and perioperative management should be strengthened. Relevant clinical workers need to continuously improve surgical methods to avoid biliary leakage complications. Additional details of all surgical operations should be provided to reduce intraoperative blood loss and to ensure strict compliance with aseptic operations. Avoiding cross-infection after surgery, achieving close follow-up and performing active individualized treatments have important clinical significance in improving the prognosis of patients.

However, the study has some limitations. First, this was a single-center study, and we did not collect clinical case information from different medical institutions. Second, due to strict screening indicators, the total number of cases was small, indicators, such as CD8 + T lymphocytes, glycated hemoglobin, FEV1/FVC ratio, portal vein tumor thrombus, microvascular invasion and other factors, cannot be further explored due to the lack of detailed data in the inquired medical record system. Additional data and a long-term follow-up are needed to better prevent postoperative infections and improve prognosis. This study was retrospective, the enrolled cases were from a single center, and the potential for selective bias could not be ignored. In future studies, we look forward to the inclusion of more treatment centers to conduct large prospective cohort studies to assess the predictors of postoperative infection.

## Conclusion

BMI, preoperative AFP, TBIL, intraoperative blood loss, and bile leakage are risk factors for postoperative infection. The prediction model established in this study can better predict the risk of infection, assist clinicians in taking effective preventive, diagnostic and treatment measures for postoperative infection in patients with PHC, and improve the prognosis of patients.

## Data Availability

The data generated by and used in the study are available from the corresponding author upon reasonable request.

## Abbreviations

PHC: Primary hepatic carcinoma
HCC: Hepatocellular carcinoma
HBV: Hepatitis B virus
HCV: Hepatitis C virus
BMI: Body mass index
NEUT: Neutrophil
ALB: Albumin
AFP: Alpha-fetoprotein
CEA: Carcinoembryonic antigen
TBIL: Total bilirubin
ALT: Alanine aminotransferase
Cr: Creatinine
PLT: Platelet count
PT: Prothrombin time
HBV DNA: Hepatitis B virus deoxyribonucleic acid
CNLC: China liver cancer staging
LASSO: Least absolute shrinkage and selection operator
C-index: Consistency index
AUC: Area under curve
DCA: Decision curve analysis
ROC: Receiver operating characteristic.
